# Dependence of Structural, Morphological and Magnetic Properties of Manganese Ferrite on Ni-Mn Substitution

**DOI:** 10.3390/ijms23063097

**Published:** 2022-03-13

**Authors:** Thomas Dippong, Erika Andrea Levei, Iosif Grigore Deac, Ioan Petean, Oana Cadar

**Affiliations:** 1 Faculty of Science, Technical University of Cluj-Napoca, 76 Victoriei Street, 430122 Baia Mare, Romania; 2 INCDO-INOE 2000, Research Institute for Analytical Instrumentation, 67 Donath Street, 400293 Cluj-Napoca, Romania; erika.levei@icia.ro (E.A.L.); oana.cadar@icia.ro (O.C.); 3 Faculty of Physics, Babes-Bolyai University, 1 Kogalniceanu Street, 400084 Cluj-Napoca, Romania; iosif.deac@phys.ubbcluj.ro; 4 Faculty of Chemistry and Chemical Engineering, Babes-Bolyai University, 11 Arany Janos Street, 400028 Cluj-Napoca, Romania; petean.ioan@gmail.com

**Keywords:** manganese ferrite, nanocomposites, amorphous silica matrix, annealing temperature, magnetic properties

## Abstract

This paper presents the influence of Mn^2+^ substitution by Ni^2+^ on the structural, morphological and magnetic properties of Mn_1−x_Ni_x_Fe_2_O_4_@SiO_2_ (x = 0, 0.25, 0.50, 0.75, 1.00) nanocomposites (NCs) obtained by a modified sol-gel method. The Fourier transform infrared spectra confirm the formation of a SiO_2_ matrix and ferrite, while the X-ray diffraction patterns show the presence of poorly crystalline ferrite at low annealing temperatures and highly crystalline mixed cubic spinel ferrite accompanied by secondary phases at high annealing temperatures. The lattice parameters gradually decrease, while the crystallite size, volume, and X-ray density of Mn_1−x_Ni_x_Fe_2_O_4_@SiO_2_ NCs increase with increasing Ni content and follow Vegard’s law. The saturation magnetization, remanent magnetization, squareness, magnetic moment per formula unit, and anisotropy constant increase, while the coercivity decreases with increasing Ni content. These parameters are larger for the samples with the same chemical formula, annealed at higher temperatures. The NCs with high Ni content show superparamagnetic-like behavior, while the NCs with high Mn content display paramagnetic behavior.

## 1. Introduction

Nanoscale materials have remarkable optical, magnetic, electrical, and catalytic properties [1,2,3,4,5,6]. The structure and composition of spinel nano-ferrites control the functional properties of magnetic nanosized materials [4,5]. Nanocomposites (NCs) are mixtures of different components at the nanometer scale, with properties that depend on the contribution of each component in the mixture [5].

Magnetic spinel ferrite (MFe_2_O_4_, where M = Zn, Co, Mn, Ni, etc.) nanoparticles are of high interest for materials science and nanotechnology, due to their high reactivity, chemical stability, and reusability [7,8,9,10]. The structure, magnetic, and electrical properties of nanosized ferrites depend upon the synthesis method, annealing temperature, as well as on the concentration, nature, and distribution of the cations between the tetrahedral (A)- and octahedral (B) sites [2]. Thus, by selecting the suitable synthesis parameters it is possible to design ferrites with the expected properties [7,11]. Particle size, shape, and enhanced surface-to-volume ratio also influence the magnetic characteristics of the nanomaterials [12]. Accordingly, the magnetization parameters are enhanced by the surface spins and spin canting [11]. Below the critical single domain, the nanomaterials have a single domain blocked state and exhibit optimum magnetic properties. In such single-domain systems, the magnetic anisotropy determines the spin alignment along the easy axis of magnetization, while the thermal fluctuations cause these spins to undergo Brownian motion along their axes [12,13,14]. As the magnetic field allows the control of the shape-memory effect, new types of microstructures may be produced by applying an external magnetic field [6]. The coercivity (*H_C_*), remanent magnetization (*M_R_*), saturation magnetization (*M_S_*), and anisotropy constant (*K*) are the main magnetic properties that determine the spinel ferrites applications [14].

The nickel ferrite, NiFe_2_O_4_, has remarkable magnetic and electrical characteristics such as high *M_S_*, permeability, resistivity, Curie temperature, and low eddy current loss [1,2,7,8,9]. NiFe_2_O_4_ has an inverse spinel structure with the Fe^3+^ ions placed equally in tetrahedral (A) and octahedral (B) sites and the Ni^2+^ ions placed in octahedral (B) sites [2,8]. Moreover, due to the magnetic moments of antiparallel spins between Fe^3+^ ions at the tetrahedral (A) sites and Ni^2+^ ions at the octahedral (B) sites of the spinel structure, NiFe_2_O_4_ displays ferromagnetic behavior [8]. The partial substitution of NiFe_2_O_4_ with magnetic divalent transition metal ions (Mn^2+^, Cu^2+^, Zn^2+^, Cd^2+^, Mn^2+^, etc.) results in exceptional properties.

The manganese ferrite, MnFe_2_O_4_, is a soft ferrite characterized by high magnetic permeability and low hysteresis losses [2]. MnFe_2_O_4_ has a spinel crystal structure with the Mn^2+^ ions occupying only the tetrahedral (A) sites, while the Fe^3+^ ions populate the octahedral (B) sites. The substitution of Ni^2+^ ions in MnFe_2_O_4_ changes its structure, magnetic, electrical, and dielectric properties [8]. When the Mn^2+^ ions are substituted by Ni^2+^ ions, Ni^2+^ ions are expected to occupy the octahedral (B) sites, while Mn^2+^ ions are randomly distributed between tetrahedral (A) and octahedral (B) sites [12,14]. Moreover, when substituting one Mn^2+^ ion with one Ni^2+^ ion, the atomic magnetic moment increases from 2 μ_B_ to 5 μ_B_ [9]. Mixed Ni-Mn ferrites present attractive magnetic properties with applications as soft and hard magnets due to their high electrical resistivity, *M_S_* and permeability, and low dielectric losses [1,9,12].

For the development of new applications, it is important to tailor the magneto-optic properties of spinel ferrites. The main routes that allow the properties tailoring for a specific application are the optimization of the synthesis parameters and selection of the optimum spinel ferrite composition [13]. Thus, the development of new ways to control the properties, especially the particle size and shape of spinel ferrites by the preparation route become of great interest [13]. The large-scale applications of nanosized spinel ferrites promoted the development of various chemical preparation methods as alternatives to solid-state reactions which produce large agglomerated particles with limited homogeneity and low sinterability [2]. Generally, the chemical methods produce fine-grained particles, but the poor crystallinity and wide particle size distribution can alter the expected properties. Moreover, the use of long reaction time and post-synthesis thermal treatment is needed [7,15,16,17,18]. Spinel ferrites are usually prepared by a standard ceramic technique that uses high temperatures and produces particles with a low specific surface [2]. Therefore, in order to obtain nanosized ferrites with high specific surface and homogeneity, alternative methods such as co-precipitation, polymeric gel, hydrothermal, micro-emulsion, heterogeneous precipitation, sono-chemistry, combustion, and sol-gel methods are used. These methods require expensive equipment, energy overriding, and high processing temperature as well as long reaction time [2,7]. The sol-gel route is the most popular way to prepare nanosized ferrites due to its simplicity, low cost, and good control over the structure and properties [10]. The microwave-assisted sol-gel method combines the advantages of microwave and sol-gel methods, being a faster, energy-saving procedure for obtaining single-phase nanopowders of high purity with accurate control of stoichiometry and capability of industrial scale-up [15,16,17,18,19,20]. The homogenous dispersion of the ferrite particles into an organic matrix also allows the production of composite materials with highly dispersed fine magnetic particles [10]. Embedding ferrites into silica (SiO_2_) matrix allows the control of the particle growth, minimizes the particle agglomeration, and enhances the magnetic guidability and biocompatibility [19].

The objective of the study was to investigate the effect of Ni content and annealing temperature on the structure, morphology, and magnetic behavior of Mn_1−x_Ni_x_Fe_2_O_4_@SiO_2_ (x = 0, 0.25, 0.50, 0.75, 1.00) NCs. The formation of ferrite and SiO_2_ matrix was investigated by Fourier transform infrared (FT-IR) spectroscopy, the formation of crystalline phases was studied by X-ray diffraction (XRD), while the shape, morphology, size, and rugosity of nanoparticles were investigated by atomic force microscopy (AFM). The variation of magnetization saturation (*M_S_*) vs. the coercive field (*H_C_*) of Mn_1−x_Ni_x_Fe_2_O_4_@SiO_2_ NCs was studied by magnetic measurements.

## 2. Results and Discussion

### 2.1. X-ray Diffraction

**Figure 1:**
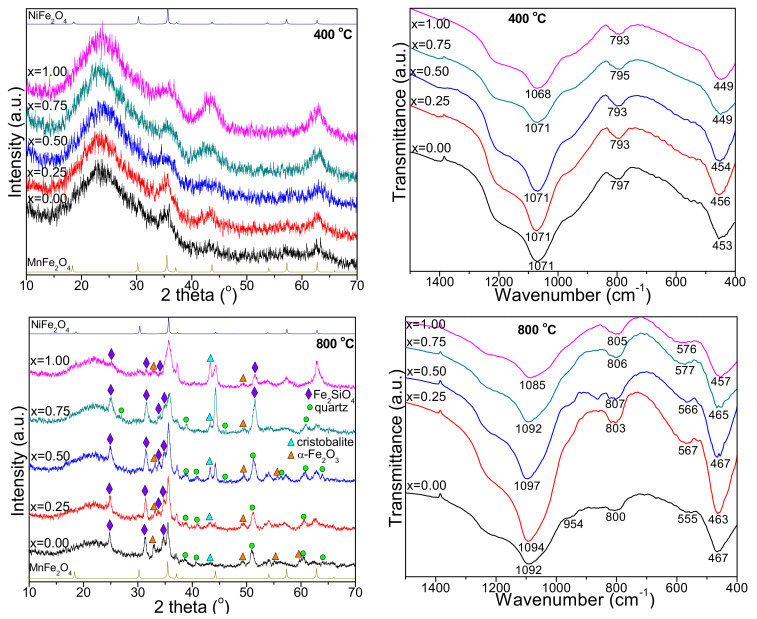

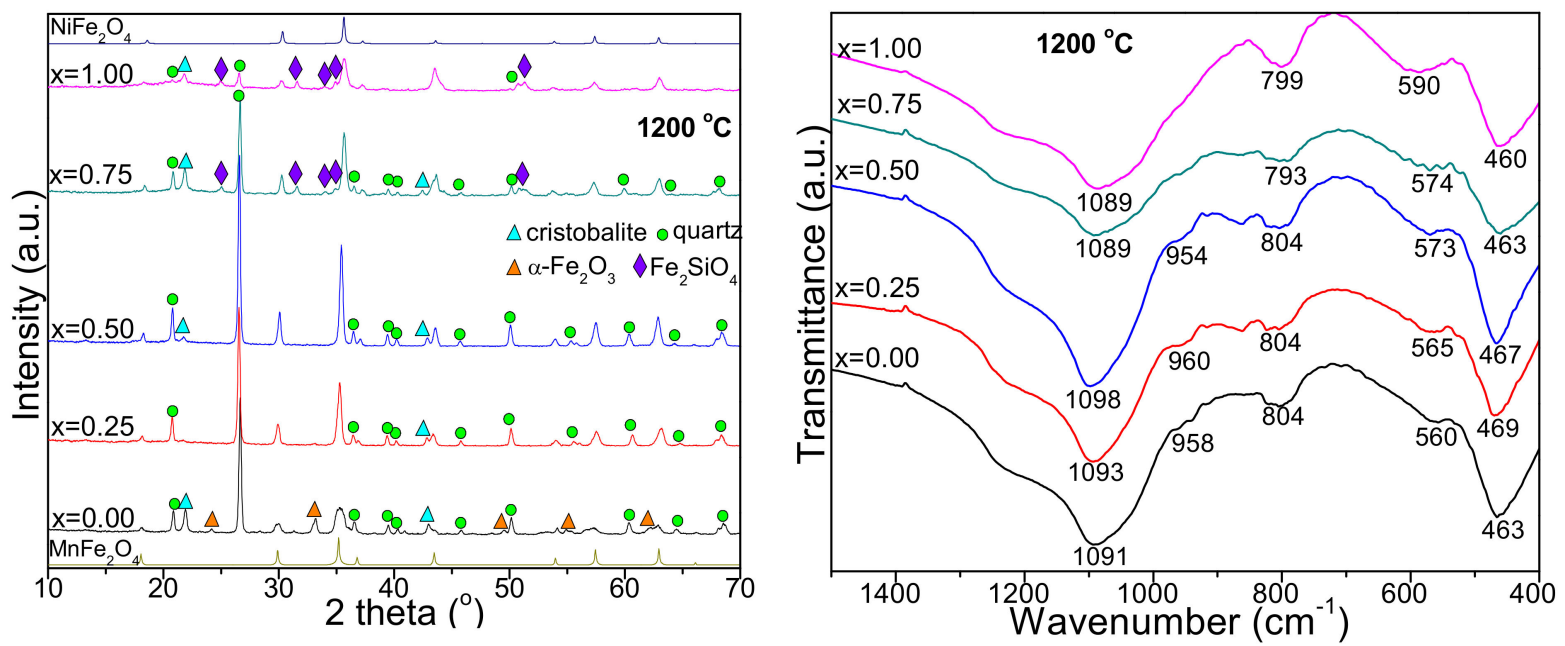
X-ray diffraction patterns and FT-IR spectra of Mn1_−x_Ni_x_Fe_2_O_4_@SiO_2_ NCs annealed at 400, 800, and 1200 °C.

The XRD patterns of Mn_x_Ni_1−x_Fe_2_O_4_@SiO_2_ NCs (x = 0, 0.25, 0.50, 0.75, 1.00) annealed at 400, 800, and 1200 °C are presented in Figure 1. At 400 °C, the baseline noise and the amorphous halo between 10 and 30° (2θ) indicate the formation of poorly crystalized ferrite, while at higher annealing temperatures, the formation of highly crystalline mixed spinel ferrites is confirmed by the sharp diffraction peaks. At higher annealing temperatures, the presence of other crystalline secondary phases is also remarked. The variation of the relative intensities and signal-to-noise ratio indicates distinct crystallinity degrees or different crystallite sizes [7].

In the case of Mn_x_Ni_1−x_Fe_2_O_4_@SiO_2_ (x = 0.00), the poorly crystallized MnFe_2_O_4_ (JCPDS card no 74-2403 [21]) is accompanied by α-Fe_2_O_3_ (JCPDS card no. 87-1164 [21]), cristobalite (JCPDS card no. 89-3434 [21], quartz (JCPDS card 85-0457 [21]) and Fe_2_SiO_4_ (JCPDS card no.87-0315 [21]) at 800 °C, and α-Fe_2_O_3_, cristobalite and quartz at 1200 °C. The diffraction peaks matching with the MnFe_2_O_4_ reflection planes (2 2 0), (3 1 1), (2 2 2), (4 0 0), (4 2 2), (5 1 1), and (4 4 0) confirm the cubic spinel structure corresponding to the space group *Fd-3m* [22]. The formation of α-Fe_2_O_3_ might be explained by the partial embedding of ferrite in the SiO_2_ matrix and the unsatisfactory annealing temperature or time needed to produce pure crystalline MnFe_2_O_4_ phase [7,8,9,19]. The formation of Fe_2_SiO_4_ could be a consequence of the reducing conditions produced by the decomposition of carboxylate precursors in the matrix pores that partially reduce the Fe^3+^ ions into Fe^2+^ ions, which react with SiO_2_ leading to the formation of Fe_2_SiO_4_ [7,8,9,10].

In the case of Mn_x_Ni_1−x_Fe_2_O_4_@SiO_2_ (x = 1.00), NiFe_2_O_4_ (JCPDS card no. 10-0325 [21]) is conveyed by α-Fe_2_O_3_, cristobalite, quartz and Fe_2_SiO_4_ at 800 °C, and cristobalite, quartz, and Fe_2_SiO_4_ at 1200 °C. The distinct formation of secondary phases of α-Fe_2_O_3_ and SiO_2_ could be attributed to the instability of Mn^2+^ ions [23,24,25]. The SiO_2_ matrix avoids the aggregation of nanoparticles through steric repulsion [24,25]. The possible oxidation-reduction reactions are also determined by the oxygen partial pressure and the presence of air during the annealing process [23,25].

In the case of Mn_x_Ni_1−x_Fe_2_O_4_@SiO_2_ (x = 0.25–0.75), at 800 °C, the ferrite is accompanied by cristobalite and quartz (x = 0.25) and cristobalite, quartz, α-Fe_2_O_3_, and Fe_2_SiO_4_ (x = 0.50 and x = 0.75). At 1200 °C, the crystalline phase of mixed Mn-Ni ferrite is accompanied by cristobalite and quartz (x = 0.25 and x = 0.50), and cristobalite, quartz, and Fe_2_SiO_4_ (x = 0.75). Some possible explanations for the formation of secondary phases could be the higher mobility of cations and the strain variation induced by the annealing process which also causes a small shift in 2θ positions and peak broadening, concomitantly with the increase of crystallite sizes [3,26].

The XRD parameters are presented in Table 1. The average crystallite size was estimated using the full width at half-maximum (w_hkl_) of the most intense (311) peak via Scherrer’s equation [11]. For the cubic structure, the lattice parameter (a) can be calculated from Miller indices (h, k, l) and inter-planar spacing (d) using the equation a = d(h^2^ + k^2^ + l^2^)^1/2^ and Bragg’s law [11]. Larger crystallite sizes were obtained at high annealing temperatures since the small nanoparticles join and form larger nanoparticles during the annealing process [12].

In NCs with a low Ni content (x = 0.25–0.50), the expansion of crystallite size is delayed, while at high Ni content (x = 0.75–1.00), the growth of crystallite size at the nucleation centers is preferred [7]. The metal ions are distributed between the tetrahedral (A) and octahedral (B) sites with oxygen as the nearest neighbor [12]. The increase of the lattice parameters at a low Ni content can be ascribed to the replacement of the smaller ionic radii Ni^2+^ (tetrahedral: 0.55Å; octahedral 0.69 Å) by the larger ionic radii Mn^2+^ (tetrahedral: 0.655 Å; octahedral: 0.80 Å). The replacement of Ni^2+^ ions by Mn^2+^ ions causes an increase of the interatomic space and, consequently, the lattice constant increase in accordance with Vegard’s law [11,22,26]. The variation of the lattice constant generates internal stress and suppresses additional grain growth during the annealing process. The difference between the theoretical and experimental values can be accredited to the approximation which considers the ions as spheres distributed in a rigid manner [1]. The obtained results are in good agreement with previous studies [8]. The crystallites are more compact in the case of NC (x = 1.00), as a Ni^2+^ ion is smaller and dissolves more easily in the spinel lattice. The decrease of unit cell volume is also observed with the introduction of smaller-sized Ni^2+^ ions in the crystal lattice [1]. There is no significant difference between the molecular weight of the obtained NCs, thus, the decrease of the unit cell volume with the increase of Ni content leads to the increase of X-ray density [1]. The X-ray density also increases with the increase of Ni content and annealing temperature. The variation of X-ray density as a consequence of small fluctuations of the lattice constant is attributed to the variation of the distribution of cations within tetrahedral (A) and octahedral (B) sites [1]. The substitution of Mn^2+^ ion generates an increase in the porosity of grains due to its greater ionic radius, the grains becoming less compact and causing an increase in particle size [26]. The hopping length (L_A_ and L_B_) between the magnetic ions in the tetrahedral (A)- and octahedral (B)- sites increases with the increase of annealing temperature and decreases with the Ni content, probably due to the higher ionic radius of Mn^2+^ in comparison to that of Ni^2+^ [1]. Furthermore, the Mn^2+^ and Ni^2+^ ions have a very low tendency for tetrahedral (A) site occupancy, while Fe^3+^ ions are unevenly divided between tetrahedral (A) and octahedral (B) sites, depending on the Ni content in the sample [1].

### 2.2. Fourier-Transform Infrared Spectroscopy

At all annealing temperatures, the FT-IR spectra show the characteristic peaks for ferrite and SiO_2_ matrix in the range of 1500–400 cm^−1^, while outside this range only the specific bands of adsorbed water are remarked (Figure 1). The specific bands of the SiO_2_ matrix appear at 1068–1098 cm^−1^ with a shoulder around 954–960 cm^−1^ attributed to the stretching and bending vibration of Si-O-Si chains, 793–807 cm^−1^ attributed to the symmetric and asymmetric vibrations of SiO_4_ tetrahedron, and 449–469 cm^−1^ attributed to the vibration of the Si-O bond that overlaps the vibration band of the Fe-O bond [19,20,27]. The high intensity of these bands suggests a low polycondensation degree of the SiO_2_ network. Additionally, to the specific bands of SiO_2_, the vibration of tetrahedral Zn-O and Ni-O bonds (555–590 cm^−1^) and the octahedral Fe-O bonds (449–469 cm^−1^) are observed [2,19,27]. These bands confirm the formation of cubic spinel structure and are in good agreement with XRD analysis [12]. The vibration band at 555–590 cm^−1^ was not observed at 400 °C, but appears at 800 and 1200 °C and increases with the increasing of annealing temperature, most probably due to the increase of the ferrite crystallization degree [19,27]. The shift of the vibration bands (555–590 cm^−1^) towards lower wavenumbers observed in samples with high Mn content is a consequence of the displacement of Fe, Mn, and Ni ions in the octahedral (B) and tetrahedral (A) sites that further leads to changes of the Fe^3+^–O^2−^ (M^3+^–O^2−^) and M^2+^–O^2−^ distances, respectively. This shift indicates a lower degree of occupancy of tetrahedral sites with Fe^3+^ ions [2].

### 2.3. Atomic Force Microscopy

As the powder samples are slightly agglomerated, the aqueous dispersion facilitates the release of free nanoparticles that are transferred onto a solid substrate as thin films prior to AFM scanning [26,28,29]. AFM images of tailored nanostructures obtained via liquid dispersion of ferrite nanoparticles were previously reported [30,31,32]. Liquid dispersion of ferrite nanoparticles allows the production of tailored nanostructures with possible application in magnetic resonance imaging [33] and 3D inkjet printing to produce ferrite nanomaterial thin films for magneto-optical devices [34].

AFM images reveal that the annealing process has a high influence on the particle size, smaller size particles being obtained at 400 °C. The particle size increases considerably with the annealing temperature, as follows: 18 nm at 400 °C, 24 nm at 800 °C, and 30 nm at 1200 °C. The smallest nanoparticles were obtained at 400 °C and the largest at 1200 °C. Mn ferrite nanoparticles are smaller than Ni ferrite nanoparticles, while the particle size of mixed Ni-Mn composition increases with the increasing of Ni content. The obtained particle sizes are slightly higher than those reported for Mn ferrite (10 nm) obtained by thermal decomposition [35] and lower than those reported for Mn ferrite annealed at high temperatures [34,36].

**Figure 2:**
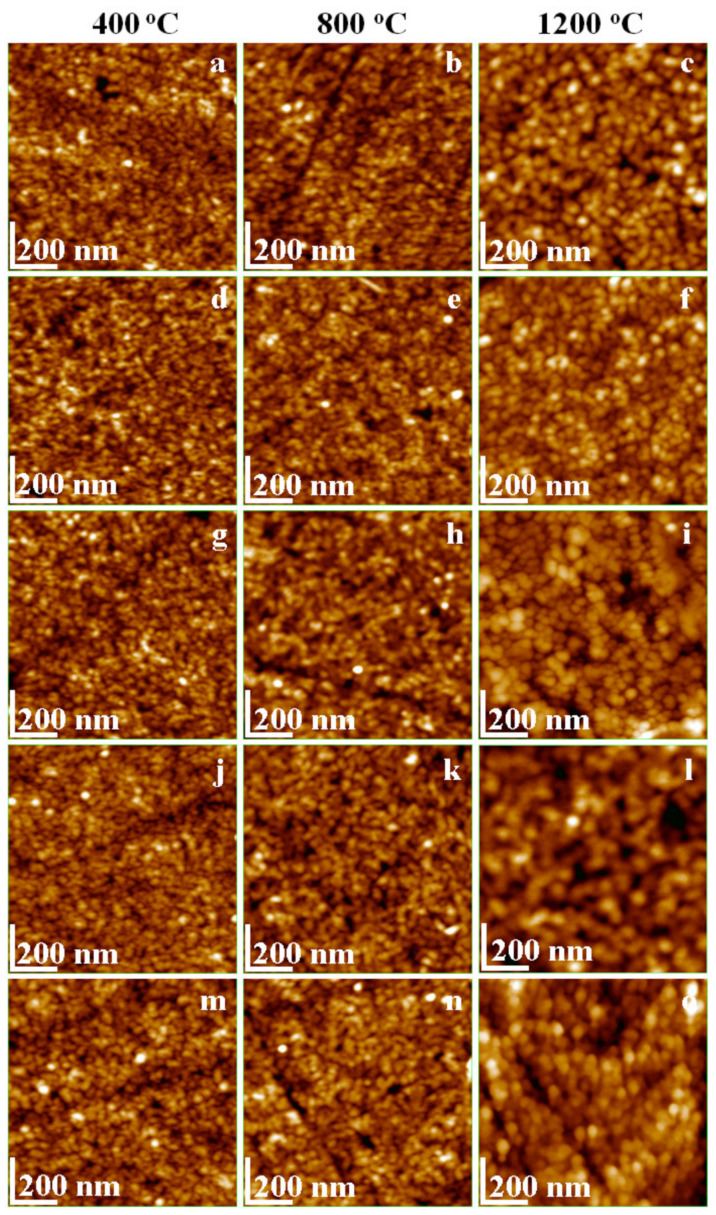
Topographical AFM images of Mn_1−x_Ni_x_Fe_2_O_4_@SiO_2_ NCs annealed at 400, 800, and 1200 °C (x = 0 (**a**–**c**), x = 0.25 (**d**–**f**), x = 0.50 (**g**–**i**), x = 0.75 (**j**–**l**) and x = 1.0 (**m**–**o**)).

The obtained NiFe_2_O_4_ particle size (Figure 2m–o) of 22 nm at 400 °C, 30 nm at 800 °C, and 58 nm at 1200 °C are in good agreement with the data reported by Tong et al. [37] and Ashiq et al. [38] for nanoparticles obtained by reverse micelle technique. The progressive replacement of the Mn^2+^ ions by Ni^2+^ ions has direct consequences on the particle size (Figure 2d–l), which progressively increases with the increase of the Ni content and the annealing temperature. In this regard, the finest nanoparticles were obtained for Mn_0.75_Ni_0.25_ Fe_2_O_4_ annealed at 400 °C, while the bigger particles for Mn_0.25_Ni_0.75_ Fe_2_O_4_ were annealed at 1200 °C. The obtained results confirmed that the modified sol-gel method resulted in very fine, highly dense, homogenous, and single-phase ferrite nanoparticles.

**Figure 3:**
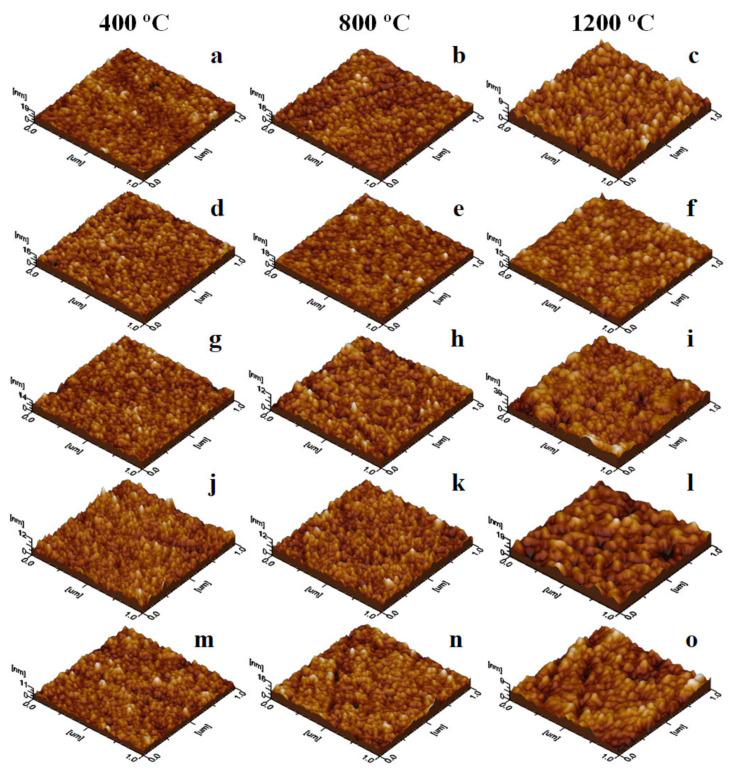
AFM 3D images of Mn_1−x_Ni_x_Fe_2_O_4_@SiO_2_ NCs annealed at 400, 800, and 1200 °C (x = 0 (**a**–**c**), x = 0.25 (**d**–**f**), x = 0.50 (**g**–**i**), x = 0.75 (**j**–**l**) and x = 1.0 (**m**–**o**)).

The nanoparticles size is slightly higher than the ferrite crystallite size estimated by the Scherrer equation, most probably due to the presence of secondary phases at high annealing temperatures. In all cases, round shape particles with a marked tendency to be adsorbed in uniform layers onto a solid substrate are remarked (Figure 3). The short deposition time allows the optimal arrangement of the particles onto the substrate and prevents their overlapping and agglomeration. Thus, the deposed thin film roughness (Table 2), depends mainly on the nanoparticle’s size. 

The smoothest thin film was obtained for the powders annealed at 400 °C, while the rougher films result from the powders annealed at 1200 °C. The tridimensional aspect is almost clogged for the ferrites with high Ni content annealed at 1200 °C due to their relatively higher size and agglomeration tendency.

### 2.4. Magnetic Properties

**Figure 4:**
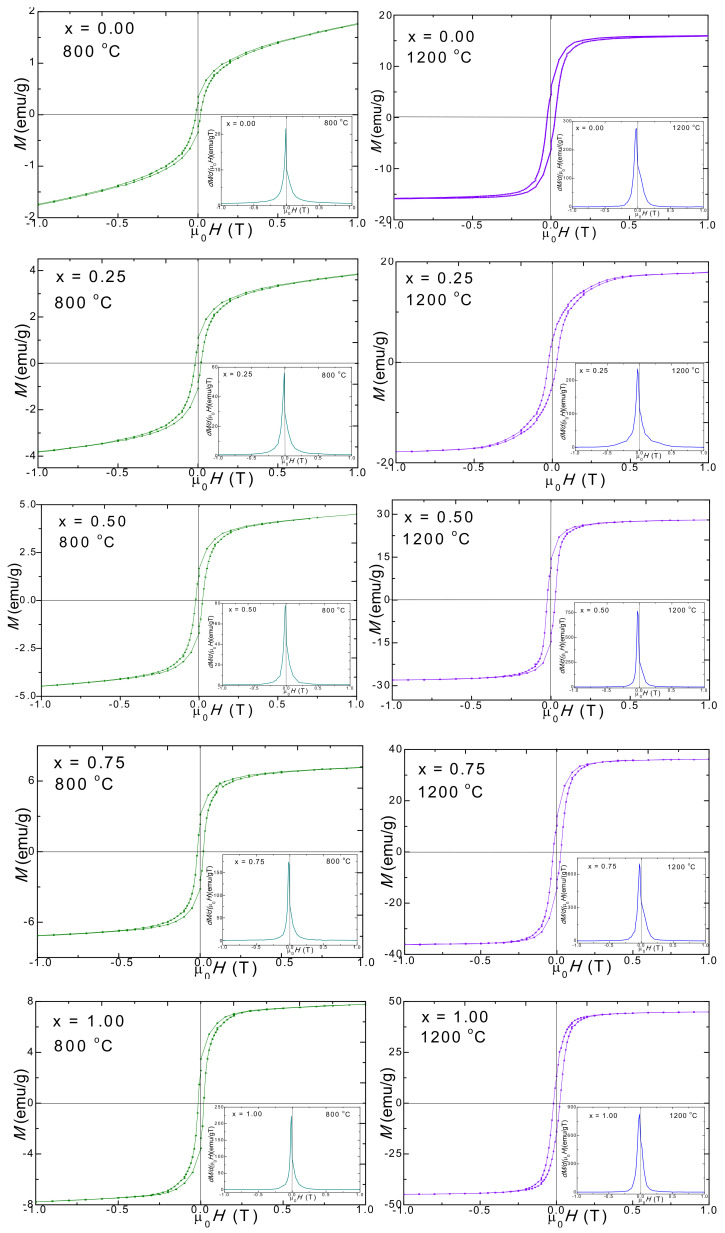
Magnetic hysteresis loops and magnetization derivative (in insets) for Mn_1−x_Ni_x_Fe_2_O_4_@SiO_2_ NCs heat-treated at 800 and 1200 °C.

Generally, the magnetic properties of the ferrites are affected by the chemical formula, by the cation distribution between the tetrahedral (A) and octahedral (B) sites of the lattice, as well as by the particle sizes and their distribution [22]. The main magnetic properties of Mn_1−x_Ni_x_Fe_2_O_4_@SiO_2_ NCs annealed at 800 and 1200 °C are displayed in Figure 4. In all cases, the hysteresis loops have a typical shape for ferrimagnetic materials. The main magnetic parameters, namely saturation magnetization (*M_S_*), remanent magnetization (*M_R_*), squareness (*S*), coercivity (*H_C_*), the magnetic moment per formula unit (*n_B_*) expressed in numbers of Bohr magnetons and anisotropy constant (*K*) extracted from the hysteresis loops are presented in Table 3.

The magnetic parameters *M_S_*, *M_R_, n_B,_* and *K* increase, while *H_C_* decreases with increasing Ni content. All the magnetic parameters are larger for the NCs with the same Ni content, annealed at higher temperatures. This behavior is different from that reported for bulk ferrites with the same chemical formula, for which an increase of the saturation magnetization was found with increasing Mn content [39]. The difference between the two systems consists in the presence of SiO_2_ coating in our samples. The largest *Ms* value was recorded for the samples with x = 1.00 (NiFe_2_O_4_@SiO_2_) with the largest particle sizes, for both annealing temperatures. *M_S_* increases almost linearly with increasing Ni content for both annealing temperatures. The *M_S_* is strongly affected by the so-called “surface spin effect” which is a result of the defects and broken chemical bonds which disrupt the parallel alignment of the magnetic moments and give rise to spin canting and spin disorder in the layer from the surface of the particles. The smaller the size of the particle, the larger the surface-to-volume ratio. Increasing the fraction of this layer will make dominant the magnetic behavior of the shell over that from the interior, and the magnetization of the smaller size particles will be reduced. In addition to this size effects, the XRD analysis also showed an increase of the hematite (which is known to have low magnetic properties) and quartz content with increasing Mn content in the samples and this can contribute additionally to the decrease of the *M_S_* value The *M_S_* value is also affected by the cation’s distribution between the tetrahedral (A) and octahedral (B) sites [22].

The increase of the *M_S_* with increasing Ni content can also suggest that in the octahedral (B) sites Fe^3+^ ions (5 µ_B_) were replaced by Ni^2+^ (2 µ_B_) ions with a smaller magnetic moment which force the Fe^3+^ ions to migrate in the tetrahedral (A) sites. This results in an inverse spinel structure since the Fe^3+^ ions are rearranged in both tetrahedral (A) and octahedral (B) sites and the antiferromagnetic interaction becomes weaker, while the ferromagnetic super-exchange interaction increases. Therefore, the normal spinel Mn ferrite is converted to a dominant inverse spinel ferrite as a result of the Mn^2+^ ions substitution by Ni^2+^ ions. This may be a consequence of the coating of Mn_1−x_Ni_x_Fe_2_O_4_ nanoparticles by the SiO_2_ matrix. Comparative *Ms* values were reported by Airimioaei et al. [2] for Ni-Mn ferrites obtained by combustion reaction, while Jessudoss et al. [22] reported higher *M_S_* and M_R_ values for Ni_1−x_Mn_x_Fe_2_O_4_ obtained by a microwave combustion reaction route. Köseoğlu reported *M_S_* between 31 and 56 emu/g at room temperature and 41–70 emu/g at 10 K [8]. Opposite to our results, the higher *M_S_* value of undoped MnFe_2_O_4_ of 66.93 emu/g steadily decreased from 64.68 emu/g (x = 0.2) to 35.43 emu/g (x= 1.0) with increasing Ni content, with NiFe_2_O_4_ showing the lower value; the linear decrease of *Ms* values of the samples could be mainly due to the difference in the magnetic moments of Mn^2+^ and Ni^2+^ ions [7].

The coercive field (*H_C_*) slightly decreases with increasing Ni content for both the samples annealed at 800 and 1200 °C (Table 3). This behavior can be attributed to the well-known dependence of the *H_C_* on the sizes of the nanoparticles in the magnetic multidomain range. In this region, the size of the nanoparticles causes them to be composed of many magnetic domains which allow an easy domain wall motion and magnetization reversal, reducing the value of the coercive field by lowering the value of the domain wall energy [1,19,28]. The *H_C_* is a measure of the magneto-crystalline anisotropy of a sample. By increasing the Ni content, the nanoparticle and the crystallite sizes also increase, leading to a decrease of the magnetocrystalline anisotropy. The *H_C_* is strongly affected by the particle’s sizes and their shape as well as by their distribution, crystallinity and magnetic domain sizes, and micro-strains induced by the SiO_2_ matrix [1]. Similarly, Airimioaei et al. reported that the *H_C_* slightly increases with increasing the amount of Mn from 37.4 Oe to 53.7 Oe for x = 0–0.5, respectively [2]. In accordance with Mathubala [7], the *H_C_* and the *M_R_* values decrease with the increase of the Ni content in MnFe_2_O_4_ lattice.

The magnetic moment per formula unit increases with the increasing of Ni content for the same reasons used to explain the behavior of the *M_S_* since the ratio of *M_S_/n_B_* is nearly constant. The increase of remanent magnetization (*M_R_)* with the increase of Ni content also needs to be correlated with the variation of the particle’s size and the related surface effects as the presence of defects and of secondary phases may act as a pinning center for the magnetic domain walls.

The anisotropy constant (*K*) reveals the energy required to rotate the magnetic moment inside the particle. The *K* increases with increasing Ni content with a factor of 1.8 for the NCs annealed at 800 °C and with a factor of 1.98 for the samples annealed at 1200 °C. A possible explanation could be the increase of magneto-crystalline anisotropy which originates in spin-orbital contribution since for MnFe_2_O_3_ the orbital quantum number is *L* = 0. Another explanation could be the presence of the spin disorder in the surface layer of the nanoparticles which needs a higher magnetic field for *M_S_*, a field that depends on the size of the particles and their distribution within the samples [8].

The squareness ratio (*S* = *M_R_/M_S_)* is a measure of how square the hysteresis loop is. A theoretical value of *M_R_*/*M_S_* lower than 0.5 indicates the presence of non-interacting uniaxial single domain particles with the easy axis being randomly oriented [1]. The *S* increases from 0.064 to 0.333 for the NCs annealed at 800 °C and from 0.246 to 0.357 for the NCs annealed at 1200 °C. Generally, the derivatives of the hysteresis loops exhibited small and broad single peaks indicating partially crystalline samples with a main magnetic phase in the presence of crystal defects. The presence of a high magnetic purity phase is indicated by the sharp peaks. The broad peaks correspond to large particle size distributions and wide coercive fields distributions. The horizontal shifts of peaks from the origin are rather small for all the samples, suggesting that the coercivities distributions are not large as a result of the magnetic interaction between the particles.

### 2.5. Potential Applications

Magnetic nanoparticles that are small enough to remain in circulation after injection and are able to pass through the capillary systems of various organs are non-toxic, well dispersed, and biocompatible and are potential candidates for biomedical applications such as cancer therapy, drug delivery, magnetic resonance imaging, or magnetic hypothermia [19,40]. The magnetic parameters of nanosized Mn_1−x_Ni_x_Fe_2_O_4_ ferrites are related to the synthesis route. The synthesized Mn-Ni ferrite nanoparticles are prospective candidates for biomedicine due to their easy synthesis process, controllable structure and size, stoichiometry control, high magnetization value, and superparamagnetic nature. Moreover, their embedding into mesoporous SiO_2_ enhances their biocompatibility and reduces their agglomeration and degradation. SiO_2_ matrix is an excellent non-toxic coating material that can create cross-linking, giving rise to an inert outer shield, avoiding the acute toxicity by ferrite inoculation [24]. However, their biocompatibility, cytotoxicity, pharmacokinetics, and other potential side effects are still underexplored. Although there are several in vitro studies that indicate the cytotoxicity through suppression of proliferation and apoptosis induction of different nanosized magnetic ferrites against human colon cancer (HT29), breast cancer (MCF7), and liver cancer (HepG2) cells, data on the Mn-Ni ferrite cytotoxicity is limited [41]. The dose-dependent cytotoxic effects of Mn_1−x_Ni_x_Fe_2_O_4_ nanoparticles against J774 E murine macrophages and U2OS human osteosarcoma was reported [42]. The effective light-to-heat conversion upon exposure of Mn-Ni ferrites to near-infrared irradiation could also be attractive for different bio-applications [42]. In order to increase their chemical stability in biological systems and for enhancing their magnetic properties different doping elements (Ni, Co, Mn, Zn, Mg, etc.) might be added [43]. Intravenous inoculation of ferrite nanosized particles can be useful as contrast agents for magnetic resonance imaging (MRI) [44]. Mn ferrite was found as a very effective MRI contrast agent in comparison with magnetite since it has large *M_S_* and high crystalline anisotropy resulting in a slower magnetic moment of relaxation [45].

## 3. Materials and Methods

### 3.1. Synthesis of NCs

Ferric nitrate nonahydrate (Fe(NO_3_)_3_∙9H_2_O) of 99.6% purity, nickel nitrate hexahydrate (Ni(NO_3_)_2_∙6H_2_O) of 99.8% purity, manganese nitrate trihydrate (Mn(NO_3_)_2_∙3H_2_O) of 100.0% purity, 1,4-butanediol (1,4BD) of 99.9% purity, tetraethyl orthosilicate (TEOS) of 100.0% purity, and ethanol of 99.9% purity were purchased from Merck (Germany) and used for synthesis without additional purifications.

The Mn_1−x_Ni_x_Fe_2_O_4_@SiO_2_ NCs (60% wt. ferrite, 40% wt. SiO_2_) were prepared by sol-gel route using Mn:Ni:Fe molar ratios of 0:1:2 (x = 0.00), 0.25:0.75:2 (x = 0.25), 0.50:0.50:2 (x = 0.50), 0.75:0.25:2 (x = 0.75) and 1:0:2 (x = 1.00). The sols were obtained by mixing the nitrate mixture with 1,4BD, TEOS and ethanol. After 4 weeks at room temperature, the gelation takes place by the formation of an SiO_2_ matrix that contains the nitrates and 1,4BD. The gels were annealed at 400, 800, and 1200 °C for 4h in air using a LT9 muffle furnace (Nabertherm, Lilienthal, Germany).

### 3.2. Characterization of NCs

The crystallinity and structure of the ferrite were investigated by X-ray diffraction recorded at room temperature, using a D8 Advance (Bruker, Germany) diffractometer, operating at 40 kV and 40 mA with CuKα radiation (λ = 1.54060 Å).

The formation of ferrite and SiO_2_ matrix was monitored using a Spectrum BX II (Perkin Elmer, Waltham, MA, USA) Fourier transform infrared spectrometer on pellets containing 1% sample in KBr.

AFM was performed by a JSPM 4210 (JEOL, Tokio, Japan) scanning probe microscope using NSC15 cantilevers (diamond-coated silicon nitride tips) with a resonant frequency of 325 kHz and a force constant of 40 N/m in tapping mode. The samples were dispersed into ultrapure water and transferred on glass slides by vertical adsorption for 30 s, followed by natural drying. Areas of 2.5 µm × 2.5 µm to 1 µm × 1 µm of dried glass slides were scanned for three different macroscopic sites.

A cryogen-free VSM magnetometer (Cryogenic Ltd., London, UK) was used for the magnetic measurements. The *M_S_* was measured in a high magnetic field up to 10 T, while the magnetic hysteresis loops were performed between −2 and 2 T, at 300 K on samples incorporated in epoxy resin.

## 4. Conclusions

The microstructure, morphology, particle size, phase composition, and magnetic properties of Mn_1−x_Ni_x_Fe_2_O_4_ (x = 0, 0.25, 0.50, 0.75, 1.00) NCs were investigated. The crystallite size (6–46 nm), X-ray density (5.050–5.249 g/cm^3^), lattice parameter (8.402–8.485 Å), and volume (593.1–606.6 Å^3^) of Mn_1−x_Ni_x_Fe_2_O_4_ increase with the increase of Ni content. The XRD patterns showed poor crystalized Mn_1−x_Ni_x_Fe_2_O_4_ at 400 °C and highly crystalline Mn_1−x_Ni_x_Fe_2_O_4_, accompanied by secondary phases of Fe_2_SiO_4_, α-Fe_2_O_3_, cristobalite, and quartz at 800 and 1200 °C. FT-IR spectroscopy confirmed the formation of the oxidic phases and SiO_2_ matrix. AFM investigations revealed round-shaped nanoparticles with sizes depending on the annealing temperature and Ni content. The magnetic properties of the NCs were strongly dependent on the chemical composition, cation distribution between tetrahedral (A) and octahedral (B) sites, as well as on the surface effects derived from the synthesis methods. For the NCs annealed at 1200 °C, *Ms* (16.4–45.7 emu/g), *M_R_*, magnetic moment per formula unit and K (2.678–5.310 erg/dm^3^) increased, while *H_C_* (260–185 Oe) slightly decreased with the increase of Ni content. The small S values indicated the presence of non-interacting single domain uniaxial particles. The magnetic parameters displayed similar behavior for the NCs annealed at 800 °C, but their variations are smaller. All the magnetic parameters increased with the annealing temperature. The obtained magnetic Mn-Ni ferrite nanoparticles are potential candidates for biomedical applications such are cancer therapy, drug delivery, magnetic resonance imaging, or magnetic hydrothermia. Despite the promising results, further studies of the biocompatibility, differential toxicity, and pharmacokinetics of nanosized Mn-Ni ferrites are required.

## Figures and Tables

**Table 1:** XRD parameters of Mn1_−x_Ni_x_Fe_2_O_4_@SiO_2_ annealed at 400, 800, and 1200 °C.

NC	Temperature, °C	Crystallite Size, nm	Lattice Parameter, Å	Volume, Å3	X-ray Density, g·cm−3	Hopping Length in A, Å	Hopping Length in B, Å
x = 0.00	400	6	8.465	606.6	5.050	3.665	2.993
	800	10	8.472	608.1	5.037	3.668	2.995
	1200	23	8.485	610.9	5.014	3.674	2.999
x = 0.25	400	8	8.441	601.4	5.114	3.655	2.984
	800	13	8.448	602.9	5.101	3.658	2.987
	1200	27	8.459	605.3	5.081	3.663	2.991
x = 0.50	400	10	8.432	599.5	5.151	3.651	2.981
	800	17	8.437	600.6	5.142	3.653	2.983
	1200	30	8.443	601.9	5.131	3.656	2.985
x = 0.75	400	12	8.416	596.1	5.202	3.644	2.976
	800	21	8.423	597.6	5.189	3.647	2.978
	1200	37	8.427	598.4	5.182	3.649	2.979
x = 1.00	400	14	8.402	593.1	5.249	3.638	2.971
	800	26	8.409	594.6	5.236	3.641	2.973
	1200	46	8.412	595.2	5.230	3.643	2.974

**Table 2:** AFM parameters of Mn_1−x_Ni_x_Fe_2_O_4_@SiO_2_ NCs.

NC’s	Temperature, °C	Height, nm	Rq Roughness, nm	Average Particle Size, nm
x = 0.00	400	19	1.15	18
	800	16	1.44	24
	1200	9	0.87	30
x = 0.25	400	16	1.16	16
	800	18	1.19	20
	1200	15	1.24	35
x = 0.50	400	14	1.08	18
	800	12	0.93	25
	1200	39	4.17	40
x = 0.75	400	12	1.05	20
	800	12	1.09	30
	1200	19	1.97	60
x = 1.00	400	11	1.08	22
	800	16	1.07	30
	1200	9	0.92	58

**Table 3:** Saturation magnetization (*M_S_*), remanent magnetization (*M_R_*), coercivity (*H_C_*), squareness (*S*), magnetic moment per formula unit (*n_B_*), and anisotropy constant (*K*) of Mn_1−x_Ni_x_Fe_2_O_4_@SiO_2_ NCs.

NC	Temperature, °C	*M_S_*, emu/g	*M_R_*, emu/g	*H_C_*, Oe	*S*	*n_B_*	K, erg/dm^3^
x = 0.00	800	4.7	0.3	200	0.064	0.194	0.590
	1200	16.4	4.2	260	0.246	0.677	2.678
x = 0.25	800	6.8	1.1	190	0.162	0.282	0.811
	1200	22.4	5.8	250	0.259	0.929	3.517
x = 0.50	800	7.8	1.7	183	0.218	0.325	0.896
	1200	29.6	13.5	240	0.456	1.232	4.461
x = 0.75	800	9.1	2.8	175	0.308	0.380	1.000
	1200	37.5	14.6	220	0.389	1.567	5.181
x = 1.00	800	10.2	3.4	166	0.333	0.428	1.063
	1200	45.7	16.1	185	0.357	1.918	5.310
